# Beyond Metabolism: The Complex Interplay Between Dietary Phytoestrogens, Gut Bacteria, and Cells of Nervous and Immune Systems

**DOI:** 10.3389/fneur.2020.00150

**Published:** 2020-03-13

**Authors:** Nicole Cady, Stephanie R. Peterson, Samantha N. Freedman, Ashutosh K. Mangalam

**Affiliations:** ^1^Department of Pathology, University of Iowa, Iowa City, IA, United States; ^2^Immunology, University of Iowa, Iowa City, IA, United States; ^3^Molecular Medicine, University of Iowa, Iowa City, IA, United States

**Keywords:** multiple sclerosis and neuroimmunology, phytoestrogen, gut microbiome, immune system, nervous system, diet

## Abstract

The human body has a large, diverse community of microorganisms which not only coexist with us, but also perform many important physiological functions, including metabolism of dietary compounds that we are unable to process ourselves. Furthermore, these bacterial derived/induced metabolites have the potential to interact and influence not only the local gut environment, but the periphery via interaction with and modulation of cells of the immune and nervous system. This relationship is being further appreciated every day as the gut microbiome is researched as a potential target for immunomodulation. A common feature among inflammatory diseases including relapsing-remitting multiple sclerosis (RRMS) is the presence of gut microbiota dysbiosis when compared to healthy controls. However, the specifics of these microbiota-neuro-immune system interactions remain unclear. Among all factors, diet has emerged as a strongest factor regulating structure and function of gut microbial community. Phytoestrogens are one class of dietary compounds emerging as potentially being of interest in this interaction as numerous studies have identified depletion of phytoestrogen-metabolizing bacteria such as Adlercreutzia, Parabacteroides and Prevotella in RRMS patients. Additionally, phytoestrogens or their metabolites have been reported to show protective effects when compounds are administered in the animal model of MS, Experimental Autoimmune Encephalomyelitis (EAE). In this review, we will illustrate the link between MS and phytoestrogen metabolizing bacteria, characterize the importance of gut bacteria and their mechanisms of action in the production of phytoestrogen metabolites, and discuss what is known about the interactions of specific compounds with cells immune and nervous system. A better understanding of gut bacteria-mediated phytoestrogen metabolism and mechanisms through which these metabolites facilitate their biological actions will help in development of novel therapeutic options for MS as well as other inflammatory diseases.

## Multiple Sclerosis and Gut Microbiota

Relapsing-remitting multiple sclerosis (RRMS) is a chronic inflammatory disease of the central nervous system (CNS), affecting ~1 million people in the US and over 2.3 million worldwide (1). Collective evidence suggests that disease results from an aberrant T-cell mediated response to myelin-derived antigens in genetically susceptible individuals. Multiple genetic and environmental factors have been implicated in the predisposition to RRMS (2). Among a number of environmental risk factors linked with MS, the gut microbiota appear to be particularly important, as highlighted by a number of recent studies reporting gut dysbiosis in RRMS patients (3–8). Although the mechanism through which the gut microbiota influences RRMS pathogenesis is unknown, diet has emerged as the strongest factor influencing the gut microbiome.

The adult human gut is colonized by a large number of microorganisms (~10^13^ bacteria). The majority of which (~90%) belong to the Firmicutes and Bacteroidetes phyla. The remainder represent Actinobacteria, Proteobacteria, and few other phyla present at very low abundance (9). The fact that only a few bacterial phyla are present in the human gut suggests that they were actively selected during human evolution. As human evolution is nutrition centric, it is hypothesized that gut bacteria capable of efficiently extracting energy from ingested plants and animal meat would provide a survival advantage. Gut microbiota composition is heavily influenced by dietary habits, with unindustrialized rural communities showing higher abundance of bacteria enriched in enzymes capable of digesting plant-based complex polysaccharides. At the same time, individuals from industrialized nations and eating a western diet rich in animal protein, fats, and simple sugars are enriched in gut bacteria containing enzymes responsible for metabolism of simple sugars, amino acids and bile acids (10). Even within a population, gut microbiota composition can be altered due to seasonal change in the food source (11). A study of Hadza hunter-gatherers of Tanzania showed this seasonal change in gut microbiome based on dietary sources, as this population rely mostly on plant based foods during rainy season but shift to plant plus meat-based diet during dry season (11). These seasonal changes in gut microbiota confirm an important role of diet (plant and meat both) in influencing the bacterial community; however, it is unclear whether one diet has advantage over other. Specifically it is unknown whether proposed pathogenic effect of meat based and/or Western diet (12) is due to meat itself or due to factors associated with industrialization/modernization such as processing of foods, increased use of antibiotics etc. (13). However, evaluation of the microbiome-mediated benefits or drawbacks of plant- vs. meat-based diets, western diet, or any other dietary interventions of interest in the scientific community are beyond the scope of this review.

Breakdown of these foods might generate key metabolites necessary for various physiologic functions of the host including development and regulation of nervous and immune systems (14). It has been clearly established that change in diet changes the composition of the gut microbiota; however the mechanisms by which this affects host physiology are slowly being understood. Non-redundant bacterial metabolites which are dependent on ingestion of certain dietary components, such as the phytoestrogens and their metabolites discussed here, are of increasing interest. As the gut microbiome plays an important role in the energy harvesting for the host, therefore any changes in the composition of the gut microbiota could have widespread effects on physiologic homeostasis and overall human health (15–18).

Our recent summary of RRMS microbiome studies across different geographical regions (USA, Japan, UK and Italy) shows enrichment or depletion of specific bacterial genera when compared to healthy controls (HC) (19, 20). One observation was loss of Bacteroidetes species, especially those of the genera *Prevotella* and *Parabacteroides* suggesting a role of these bacteria in RRMS [Table 1; (3–8)]. Our group (19) and others (3–5) reported a lower abundance of *Prevotella* in fecal samples of RRMS patients compared to HC. Additionally, treatment with disease modifying therapies (DMT) led to a higher abundance of *Prevotella* in RRMS patients than in untreated patients (5, 20). Further, Cosorich et al. also reported lower level of *Prevotella* when analyzing duodenal biopsies from RRMS patients with active disease compared to HC (7). Another Bacteroidetes genus, *Parabacteroides* has been reported to be at lower abundance in adult RRMS patients when compared to HC (4, 8). We observed reduced abundance of *Parabacteroides* in RRMS patients vs. HC from the Midwestern United States (4). Similarly, Cekanaviciute et al. reported that *Parabacteroides distasonis* is at lower abundance in treatment naive RRMS patients from the US west coast than HC, suggesting that higher level of *P. distasonis* may protect against RRMS (8). Further, we also observed a lower abundance of the phytoestrogen metabolizing bacteria *Adlercreutzia (equolifaciens)* in RRMS patients compared to HC (4) and *Adlercreutzia* was also reported to be increased in germ-free (GF) mice transplanted with fecal matters from HC compared to mice receiving fecal transplant from RRMS patients (21).

**Table 1:** Comparison of adult MS microbiome studies.

**MS Microbiome Study # samples Tissue (Country) *[Reference]***	**Lower abundance in MS patients vs. HC**	**Increased abundance in MS patients after treatment**
RRMS (*n* = 31) HC (*n* = 36) Fecal (USA) Chen et al. (4)	***Prevotella**, **Parabacteroides**, **Adlercreutzia**, Collinsella, Lactobacillus*	
RRMS (*n* = 60) HC (*n* = 43) Fecal (USA) Jangi et al. (5)	*Butyricimona, Prevotella, Parabacteroides*	***Prevotella Sutterella***
RRMS (*n* = 20) HC (*n* = 40) Fecal (Japan) Miyake et al. (3)	*Bacteroides, Fecalibacterium, **Prevotella***, *Anaerostipes, Clostridium, Sutterella*	
RRMS (*n* = 30) HC (*n* = 14) Fecal (UK) Castillo-Alvarez et al. (20)		***Prevotella***
RRMS (*n* = 71) Fecal (USA) Cekanaviciute et al. (8)	***Parabacteroides distasonis***	
RRMS (*n* = 19) HC (*n* = 17) Mucosa (Italy) Cosorich et al. (7)	***Prevotella***	

*Bolded microbes are phytoestrogen metabolizing bacteria*.

Thus, these studies indicate that loss of Bacteroidetes genera *Prevotella* and *Parabacteroides* might play a role in the predisposition and/or exacerbation in RRMS. As MS is an inflammatory disease where balance between pro- and anti-inflammatory responses are shifted toward inflammatory responses, it is reasonable to hypothesize that bacteria depleted in MS were involved in induction/maintenance of anti-inflammatory responses. More discussion relating to the possible mechanism of this protective role through induction of immunoregulatory cells will be discussed in this review under Phytoestrogens and Immune Cells.

Conversely, Firmicutes such as *Akkermansia, Dorea*, and *Archaea-Methanobrevibacter* were more abundant in stool from RRMS patients (4, 5, 8), suggesting that these gut microbes might have pro-inflammatory effects. This increased abundance could reasonably contribute to the induction and/or maintenance of pro-inflammatory cells in the gut, thus influencing or contributing to a systemic inflammatory state consistent with RRMS. However, *Akkermansia* had been shown to have anti-inflammatory effects in obesity and diabetes due to their ability to produce short-chain fatty acids (SCFA) (22). Similarly, *Dorea* has been suggested to be anti-inflammatory based on the observation that patients with pouchitis and Crohn's disease-like have lower abundance of *Dorea* (23) Overall, the mechanisms through which these bacteria might induce inflammation and the factors which may influence this are not well-understood and beyond the scope of this review.

**Figure 1:**
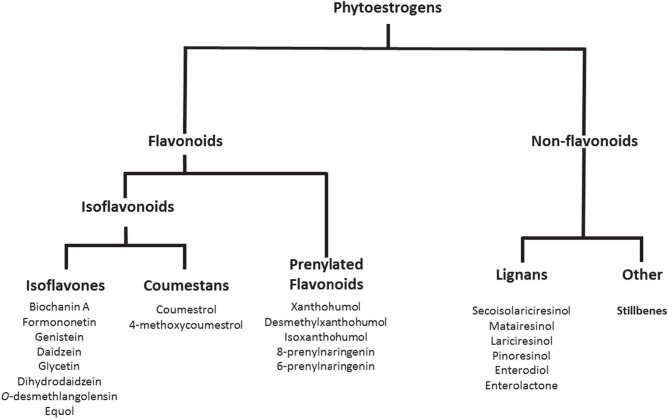
Phytoestrogens and their metabolites generated with the help of gut bacteria. Copyright © 2019 American Chemical Society. All Rights Reserved.

Phytoestrogens are compounds produced naturally in plant foods such as legumes, soybeans, beans, nuts, flax seeds, sesame seeds, hops, and other plants (Figure 1). They are known to have estrogenic/antiestrogenic, antioxidant, and anti-inflammatory effects, among others (24). It is important to highlight that the role of phytoestrogens in the cancer field has been studied extensively; however, their significance in inflammatory autoimmune diseases is less understood. *Prevotella, Parabacteroides, and Adlercreutzia* are known to metabolize phytoestrogens and produce secondary molecules such as equol, enterolactone, and secoisolariciresinol (Table 2). These bacteria can also metabolize fibers to produce SCFAs(as reviewed in Freedman et al. (62). Importance of the gut microbiota in MS has been studied extensively in its animal model experimental autoimmune encephalomyelitis (EAE). The suppression of EAE disease in GF mice on fecal transfer from HC and exacerbation of disease on fecal transfer from MS patients supports a critical role of gut microbiota in MS (8, 21). Gut bacteria have been shown to influence disease through modulation of multiple metabolic pathways such as short-chain fatty acid (63–65), tryptophan (62, 66), and phytoestrogen metabolism (66). As discussed before, we and others have reported the loss of bacteria involved in phytoestrogen metabolism (62). Through metabolism of phytoestrogens, these microbes may play an important role in the regulation of inflammation; thus, we hypothesize that reduced abundance of phytoestrogen metabolizing bacteria in the gut would influence the inflammation and demyelination in RRMS (4). Therefore, in this review we will focus on the importance of phytoestrogen metabolism by the gut microbiota, as well as on the effect of this phenomenon on the host physiology. We will discuss in detail: the mechanisms whereby gut bacteria metabolize phytoestrogens into structurally and functionally distinct metabolites; the ability of such metabolites to modulate various physiological processes, such as immune and neuronal/glial cell activity; and the ability of the metabolites to modulate disease in animal models of MS.

**Table 2:** Phytoestrogen metabolizing gut bacteria.

**Metabolite**	**Strain**	**Reference**
Daidzein	Eubacterium limosum	(25)
	*E. coli strain HGH21*	(26)
	*Bifidobacterium animalis*	(27)
	*Bifidobacterium longum-a*	(27)
	*Bifidobacterium pseudolongum*	(27)
	Strain HGH6	(26)
Genistein	*Eubacterium limosum*	(25)
	*HGH21*	(26)
	*Strain HGH6*	(26)
DHD	*Asaccharobacter celatus*	(28)
	*Coprobacillus* sp. *MRG-1*	(29)
	*Lactococcus garvieae (Lactococcus* sp. *20–92)*	(30)
	*Strain HGH6*	(31)
	*Strain TM-40*	(32)
Equol	*Adlercreutzia equolifaciens FJC-B9T*	(33)
	*Asaccharobacter celatus*	(28)
	*Asaccharobacter celatus AHU1763*	(34)
	*Bacteroides (parabacteroides) distasonis#*	(35)
	*Bacteroide ovatus spp*.	(36)
	*Bifidobacterium spp*.	(37)
	*Bifidobacterium* spp.	(27)
	*Coriobacteriaceae* sp *MT1B9*	(38)
	*Eggerthella* sp. *YY7918*	(39)
	*Enterococcus faecium EPI1*	(40)
	*Finegoldia magna EPI3*	(41)
	*Lactobacillus mucosae EPI2*	(34)
	*Lactobacillus* sp. *Niu-O16*	(42)
	*Lactobacillus rhamnosus JCM 2771*	(43)
	*Prevotella veroralis*	(35)
	*Ruminococcus productus*	(44)
	*Ruminococcus productus spp*.	(36)
	*Slackia equolifaciens DEZ*	(45)
	*Slackia isoflavoniconvertens HE8*	(46)
	*Slackia* sp. *NATTS*	(47)
	*Strain Julong 732*	(41)
	*Streptococcus intermedius spp*.	(36)
	*Veillonella spp. EP*	(31)
	*Veillonella spp. EP*	(48)
5-hydroxy-equol	Coriobacteriaceae sp MT1B9	(38)
	Slackia sp HE9	(46)
ODMA	*Clostridium* spp. HGHA136	(31)
	Eubacterium ramulus	(48)
	Eubacterium ramulus wK1	(48)
	Strain SY8519	(39)
Secoisolariciresinol	*Bacteroides fragilis*	(49)
	*Bacteroides (Parabacteroides) distasonis^#^*	(49)
	*Bacteroides ovatus*	(49)
	*Bifidobacterium bifidum WC 418 and WC 421*	(50)
	*Bifidobacterium catenulatum ATCC 27539*	(50)
	*Bifidobacterium longum* subsp. *infantis ATCC 15697*	(50)
	*Bifidobacterium longum* subsp. *longum WC 436 and WC 439*	(50)
	*Bifidobacterium pseudocatenulatum WC 401, WC402, WC402 and WC 407*	(50)
	*Butyrivibrio fibrosolvens*	(51)
	*Butyrivibrio proteoclasticus*	(51)
	*Clostridium ramosum*	(50)
	*Clostridium cocleatum*	(49)
	*Clostridium* sp. *SDG-Mt85-3Db*	(49)
	*Eggerthella lenta*	(52)
	*Prevotella albensi*	(51)
	*Prevotella brevis*	(51)
	*Prevotella breyantii*	(51)
	*Prevotella ruminicola*	(51)
Dihydroxyenterodiol	*Butyribacterium methylotrophicum*	(49)
	*Eubacterium callanderi*	(49)
	*Eubacterium limosum*	(49)
	*Peptostreptococcus productus*	(49)
	*Clostridiaceae bacterium END-2*	(53)
Enterodiol	*Clostridium scindens*	(49)
	*Eggerthella lenta*	(49)
	*Eubacterium* sp. *ARC-1*	(54)
	*Enterococcus faecalis*	(55)
Enterolactone	*Strain ED-Mt61/PYG-s6*	(49)
	*Eubacterium* sp. *ARC-1*	(54)
	*Eggerthella* sp. *SDG-2*	(54)
	*Enterococcus faecalis*	(55)
	*Ruminococcus* sp. *END-1*	(54)
	*Clostridiaceae bacteriumEND-2*	(54)
8-prenylnaringenin	*Eubacterium limosum*	(56)

Adapted from Lopes et al. (57), Yoder et al. (58), Sánchez-Calvo et al. (59), Setchell et al. (60), Rafii et al. (61)

# *Parabacteroides was classified as Bacteroides in old nomenclature*.

## Phytoestrogen Metabolites in the Gut

**Figure 2:**
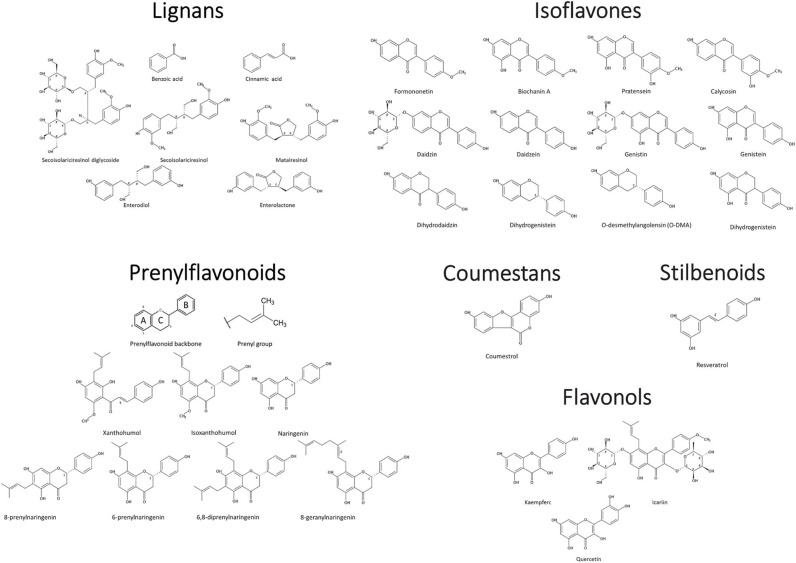
Phytoestrogen structure and classification. Chemical Structures Copyright © 2019 American Chemical Society. All Rights Reserved.

Phytoestrogens can be categorized, based on their structures, as flavonoids or nonflavonoids (Figure 1). Flavonoids are phenolic compounds with a basic structure consisting of 3 rings (denoted A, B, and C) comprised of 15 carbon atoms arranged in two aromatic rings connected by a 3-carbon bridge (67). These rings give them structural similarity to estradiol and the ability to mimic the function of estrogen. Further subclassification such as those discussed in this review (coumestans, prenylflavonoids, and isoflavones) are distinguished according to structural differences in the connection between the B and C rings, as well as the degrees of saturation, oxidation, and hydroxylation of the C ring [Figure 2; (67)]. Non-flavonoids consist of phenolic acids in either C6-C1 (benzoic acid) or C6-C3 (cinnamic acid) conformations, stilbenes, and lignans, the latter being the primary class implicated in microbiota-induced influence of human health (68).

### Coumestans

The primary molecule studied in the coumestan family is coumestrol. In addition to the classical flavonoid structure, coumestrol has a furan ring in the junction between the C and B rings (69) and one hydroxyl group each at the C4 and C7 carbons, similar to the structure of estradiol (57). These moieties confer the ability to bind mammalian estrogen receptors (70) and provide free radical scavenging properties (69, 71), thus suggesting that coumestans may provide protection against breast, prostate, and ovarian cancers (72–74).

*In vitro* treatment of MCF7:WS8 (estrogen sensitive) and MCF7:5C (estrogen deprived) breast cancer cells with coumestrol had anti-proliferative and pro-apoptotic effects, respectively, which depended on estrogen receptor alpha (ERα) signaling (75). Similar observations were made in triple-negative breast cancer cells, but through the estrogen receptor (ER)-independent Bax/Bcl-2 pathway (76). Other coumestans have not been well-characterized in relation to their estrogenic activity. Furthermore, as coumestrol has an affinity for mammalian estrogen receptors reportedly only 10–20 times lower than 17β-estradiol (77), it is one of the more potent phytoestrogen compounds. Taken together, coumestrol and potentially novel coumestans or coumestan metabolites may prove to be an interesting target for study in neurological diseases such as MS.

### Prenylflavonoids

Prenylflavonoids are characterized by prenylated side chains on the flavonoid backbone, generally at the 6, 8, 3, and/or 5 positions (78). Prenyl chains come in numerous forms, notably 3,3-dimethylallyl substituent, geranyl, 1,1-dimethylallyl, and is moieties (79). The most common prenylflavonoids in the human diet are xanthohumol and isoxanthohumol, both of which are found in hops, beer, and an increasing number of dietary supplements (80, 81). A systematic review of the prenylflavonoid literature in 2014 revealed an association of these molecules with a variety of biological effects (including cytotoxicity, particularly against tumor cells), as well as antibacterial effects (their primary function in the plant species in which they were originally identified) (79). Prenylflavonoids also modulate the functions of many enzymes; e.g., they inhibit the activities of cholinesterase and aldose reductase (AR), and they enhance those of alcohol- and aldehyde dehydrogenases (79). Like coumestans, they have also been shown to have anti-oxidant activity, including strong radical scavenging properties. Finally, xanthohumol metabolite 8-prenylnaringenin's (8PN) *in vitro* estrogenic activity was shown to be stronger than that of other phytoestrogens, including coumestrol, genistein, daidzein; as well as its precursors xanthohumol, isoxanthohumol, naringenin, and related compounds 6-prenylnaringenin, 6,8-diprenylnaringenin and 8-geranylnaringenin (82–85).

Numerous studies have described the processing of prenylflavonoid compounds, xanthohumol and isoxanthohumol, by human liver microsomes (81, 86–88). Nikolic et al. also characterized the processing of 8PN in human liver microsomes (80), which is thought to be more potent ligands of ER than original plant compounds. Recently, however, *Eubacterium limosum* was also shown to convert isoxanthohumol into 8PN and to demethylate some isoflavonoids (89). Furthermore, both the yeast species *Pichia membranifaciens* (ATCC 2254) and the fungal species *Cunninghamella echinulata* (NRRL 3655) were found to metabolize 8PN (90, 91). The additional metabolism by gut microbiota gives potential for more estrogenic compounds to be bioavailable. Thus, biological relevance of this metabolite may be greatly altered when considering the actions of commensal species, especially if prenylflavonoids follow similar patterns of absorption to other dietary polyphenols and 80–90% of consumed compound is potentially available to colonic bacteria (92). Furthermore, additional metabolites of 8PN produced with the help of gut bacteria, could exist and have various biological effects (80).

### Lignans

Although isoflavones are the best studied phytoestrogens, lignans are significantly more prominent in the Western diet (93–95). Dietary lignans are found at high levels in seeds such as flax and sesame seeds, and more broadly in cereals, fruits, and vegetables at moderate to high levels (58, 96). These non-flavonoid compounds are distinguished by a unique coupling of phenylpropanoid units at their 8' positions. The two major dietary lignans, secoisolariciresinol (or secoisolariciresinol-diglycoside) and matairesinol, give rise to the metabolites enterodiol and enterolactone. When first discovered, these metabolites were thought to originate from the ovaries, but further study in antibiotic-treated and germ-free rats indicated that intestinal bacteria are required for the production of both enterodiol and enterolactone (97). This was later confirmed in humans as well (98). The mechanism by which gut microbiota enzymes metabolize lignans is well-established (52, 99–101). From secoisolariciresinol-diglycoside, hydrolysis of the sugar moiety takes place first, followed by subsequent dehydroxylation, and demethylation to produce enterodiol (99, 102). Enterodiol can be further oxidized to enterolactone (99, 102). Similarly, matairesinol is dehydroxylated and demethylated to form enterolactone directly (102).

Both of these lignan metabolites have far greater biological effects than their precursors. The identification of both compounds during pregnancy and the cyclic pattern of excretion in females during menstrual cycle (established in both humans and monkeys) have physiological implications possibly related to interactions with ER, though whether these are estrogenic or antiestrogenic is unclear (103). Furthermore, both compounds are inhibitors of enzymes involved in steroid metabolism such as aromatase, 5 α-reductase, and 7β-hydroxysteroid dehydrogenase (103–106). Potential anticancer activity via antiestrogenic or antioxidant activity has been long proposed and well-studied (107–112). While parent compounds certainly have some physiological effects (113–117), the metabolism by microbiota serves to greatly alter and/or enhance the function of lignans.

### Isoflavones

Isoflavones are low molecular-weight compounds derived from plants with hydroxyl groups in the C4 and C7 positions, similar to coumestans discussed above, and estradiol (57). Isoflavones are among the most studied dietary phytoestrogens, and they are abundant in soybeans and soybean products, as well as in several other legumes (96, 118). Formononetin and biochanin A are processed, via either intestinal glucosidases (96) or enzymes in hepatic microsomes (118), to the more estrogenic compounds daidzein and genistein. Furthermore, daidzein, and genistein can be found directly in foods, generally in their glycoside forms bound to a sugar moiety. For gut absorption, isoflavone glycosides (e.g., daidzin and genistin), must be further processed into the aglycone form which lacks this sugar moiety (119, 120). Once hydrolyzed, they are readily absorbed and are detectable in plasma, urine, and feces (120). In rats, the aglycone forms of genistein and daidzein were detectable in plasma as soon as 3 min after an oral dose was administered (121). In a separate experiment in which glycoside forms of the same isoflavones were administered, detection was much slower because processing by intestinal β-glucosidases (expressed at highest levels in the duodenum) was required for absorption (121).

Isoflavones genistein, dihydrogenistein, and equol are proposed to bind ERβ with nearly the same, or slightly lower, affinity as 17β-estradiol, while affinity for ERα is generally weaker (122, 123). Isoflavones provide several benefits, including: antioxidant and antiangiogenic effects (57, 124–126); protection against breast cancer (127); and prevention of several menopause-related conditions (128–131). These effects are all thought to be mediated through the activation of ERα and/or ERβ, though in many cases the exact mechanisms are unknown.

The metabolism of daidzein to equol or *O-*desmethylangolensin (*O*-DMA) is entirely dependent on one or more bacterial strains in the gut, including but not limited to *Adlercreutzia equolifaciens, Eggerthella* sp., and *Slackia isoflavoniconvertens* (33, 132) isolated from humans, A*saccharobacter calatus* and *Enterorhabdus musicola* identified in mice [Table 2; (133)]. These bacteria contain a specific set of enzymes, including daidzein reductase, dihydrodaidzein reductase, and tetrahydrodaidzein reductase required to metabolize daidzein into equol and/or *O-*desmethylangolensin (ODMA) (134). Biologically, it is *S*-equol (S-EQL) that is found in mammals and therefore has been the target of most research, whereas *R*-equol (R-EQL) could only be synthetically produced. However, more recently racemic mixture has been detected following synthesis by Lactococcus strain 20–92 and Eggerthella strain Julong 732 (135). The importance of *O*-DMA in human physiology is not well-understood and further research is needed to determine its significance to human health.

## Phytoestrogens and Processing in the Gut

Gut microbes are thought to play an essential, non-redundant role in the metabolism of phytoestrogens in humans. This notion is supported by the fact that both GF mice on a soy-based diet and newborn infants up to 4 months of age (both of which lack diverse microbiota) lack equol (97, 136, 137). Additionally, culturing of human fecal matter from equol-producing individuals with soy or daidzein resulted in the formation of S-EQL (138, 139), and the inclusion of antibiotics in these cultures resulted in inhibition of equol production (139). Although the importance of intestinal bacteria in S-EQL production is well-established, the bacterial enzymes required and the microbes which contain them are slowly being characterized. Further research in this area and characterization of these bacteria in diseases vs. healthy states may provide important insight into mechanisms behind negative correlations observed with numerous diseases (e.g., obesity, breast cancer) in populations which consume high amounts of soy.

The majority of S-EQL is produced by conversion of daidzein, via enzymes derived from gut bacteria. However, daidzin, which is present in plant-based foods, must first be hydrolyzed into the bioactive aglycon form, daidzein. This hydrolysis step is catalyzed by β-glucosidase in the brush border membrane of the proximal intestine (140). As conjugated forms (glucosides) cannot cross intestinal epithelial cells, hydrolysis is a critical step in the formation of bioactive isoflavone metabolites. Three enzymes are required to metabolize daidzein into *S*-EQL and ODMA: daidzein reductase (DHNR), dihydrodaidzein reductase (DHDR), and tetrahydrodaidzein reductase (THDR) (46). Similar mechanisms might be involved in the digestion of other phytoestrogenic compounds and in the production of small metabolites.

## Phytoestrogen Receptors

The downstream effects of phytoestrogens are thought to be mediated, in part, through estrogen receptors, which are expressed widely, including in the cells of the immune and nervous systems (141, 142). Phytoestrogens and their metabolites can interact with the prototypic estrogen receptors, ERα and ERβ, effecting changes in cell physiology through modulation of transcription and gene expression (Table 3). Alternatively, phytoestrogen can also signal through the G-protein coupled estrogen receptor (GPER), which allows for more rapid and dynamic regulation of cell processes because the mechanisms are predominantly non-genomic (143–145). However, the majority of research in phytoestrogen signaling has focused on signaling through ER receptors and their ability to activate ER receptors compared to the natural ligand 17β- estradiol. Individual phytoestrogen metabolites have been proposed to have higher affinity for one ER over the other. For example, genistein and daidzein have significantly higher affinity for ERβ than for ERα (141). As ERα is the predominant estrogen receptor on the cells of the immune system (142) either the signaling pathways used by internal estrogens and phytoestrogens/phytoestrogen metabolites differ slightly; or ER-independent pathways such as GPER signaling might play a significant role in phytoestrogen-mediated modulation of immune cells. This may mean that greater shifts in phytoestrogen availability are needed to potentiate a change in signaling; however, additional research is needed to better understand the mechanisms of action of phytoestrogens and their metabolites in regard to receptor binding and the signaling pathways required for their biological activities, especially in the context of cells of immune and nervous systems.

**Table 3:** Estrogen Receptor Expression on Immune Cells.

**Cell Type**	**Human**		**Mouse**			
	**ERα**	**ERβ**	**ERα**	**ERβ**	**GPER**	**Reference**
CD4+ T-cell	Yes	Yes	Yes		Yes	(142, 143)
CD8+ T-cell	Yes	Yes			Yes	(142, 143)
B-cell	Yes	Yes	Yes	Yes	Yes	(142, 143)
NK Cell	Yes	Yes	Yes	Yes		(142)
Macrophages			Yes	No	Yes	(142, 143)
Monocyte-derived DC	Yes	Yes				(142)
Bone marrow-derived DC			Yes	Yes		(142)
Splenic DC			Yes	No		(142)
Plasmacytoid DC	Yes	Yes	Yes			(142)
CNS inflammatory DC				Yes		(142)

## Phytoestrogens and Neurons

After phytoestrogens are metabolized in the gut and transported to the liver, they may have systemic effects (52). With regard to the CNS, an organ system in which ERs are widely expressed, phytoestrogen metabolites have been found to have direct neuroprotective effects, based on both *in vitro* and *in vivo* studies (in animal models). Phytoestrogens can exert neuroprotective effects by attenuating toxic insults to neurons. For example, several studies showed that toxin-induced plasma-membrane damage was reduced in neurons treated *in vitro* with genistein and daidzein (146). In another study, such treatment resulted in neural-cell proliferation and improved cell viability (147). Furthermore, quercetin and kaempferol (phytoestrogen flavonoids) prevented neuronal cell death in the context of oxidative stress (148). Phytoestrogens can also exert neuroprotective effects by attenuating microglial mediated inflammatory responses; one study found that formononetin, daidzein, pratensein, calycosin, and irilone attenuated LPS-induced proinflammatory cytokine production by microglia (149).

Several *in vivo* studies have documented neuroprotective effects of phytoestrogens in the diet. The synaptic density was much greater in rats fed either a daidzein- plus genistein-based diet or a soy-enriched diet, compared to rats on either a standard chow or diet lacking soy (150). Furthermore, rats fed a diet containing soy-derived phytoestrogens exhibited improved learning and memory compared to rats on a control diet (151). Additionally, pretreatment with phytoestrogens protected mice from neurotoxicity in the CNS following 1-methyl-4-phenyl-1,2,3,6-tetrahydropyridine (MPTP)-induced Parkinson's Disease (PD) (152). These studies suggest that phytoestrogens might alter the structure and/or the function of both healthy and diseased neurons.

The mechanism underlying phytoestrogen-induced neuroprotective effects might be related to estrogen receptor agonistic activity. Estrogen replacement therapy (ERT) has been shown to improve CNS function, especially in Alzheimer's disease, by preventing oxidative stress and the formation of amyloid plaques (153). Indeed, genistein, daidzein, and zearalenone stimulate ERα- and ERβ-dependent transcription of genes that contain estrogen response elements (EREs) in their promoters (154). However, phytoestrogens have been shown to be 100–1,000 × less potent than 17β-estradiol, but they can also target GPR-30, a GPER on the plasma membrane of number of cells in a variety of tissues (155). However, further research is needed to determine the significance of the GPER receptor in phytoestrogen mediated signaling.

## Phytoestrogens and Immune Cells

Phytoestrogens have varied effects on immune system function which have been summarized in Table 4. These effects are most often anti-inflammatory and protective in nature. In the case of the adaptive immune system, studies have shown that genistein and other isoflavones can suppress lymphocyte proliferation, allergic responses, and antigen-specific immune responses in both T- and B-cells (156, 158, 162–165). However, genistein has also been shown to enhance both the cytotoxic activity of CD8 T-cells, and the production of cytokines by T-cells more generally (156–161). Notably, this largely mirrors the effects of estrogens on these cells. One study by Kojima et al. showed that various phytoestrogens enhance gene expression mediated by retinoic-acid-receptor-related orphan receptor (ROR) γ and α in T-lymphoma cells, leading to increased expression of IL-17. Others have shown that when activated T-cells are treated with formononetin, daidzein, or equol, the levels of IL-4 expression increase (159). Collectively these studies suggest that phytoestrogens can interact with the T-cell compartment to induce various responses that may improve disease outcomes. Although the mechanisms leading to these actions are not well-understood, it has been suggested that either enhancement or inhibition of the NF-kB pathway could contribute, especially to cytokine responses.

**Table 4:** Summary of effects of phytoestrogen compounds on various cell types.

**Cell Type**	**Results**	**Compound**	**Model**	**References**
T-cell	Enhanced CD8+ T-cell cytotoxicity	Genistein	Genistein treatment of mice with B16 melanoma	(156, 157)
	Increased cytokine expression and production	Genistein, formononetin, daidzein, equol	*Ex vivo* stimulation of cells from treated mice; *In vitro* gene expression studies	(158–161)
	Enhanced RORγ and RORα expression	Genistein, formononetin, daidzein, biochanin A	*In vitro* gene expression in Jurkat and CHO-K1 cells	(160)
	Suppressed proliferation	Genistein	*Ex vivo* stimulation of cells from genistein treated mice	(156, 158)
	Suppressed antigen-specific responses	Genistein	*In vivo* in immunized/sensitized mice	(158, 162–164)
B-cell	Decreased antigen specific IgE, IgG2a, IgG3, and IgG1	Isoflavones, coumestrol	*In vivo* in peanut antigen sensitization model and experimental autoimmune thryoiditis	(162–165)
NK cell	Enhanced cytotoxicity	Genistein	*Ex vivo* stimulation of NK cells from treated mice or rats	(156, 157, 163, 166)
	Increased degranulation	Genistein, daidzein	*In vitro* co-culture with treated, activated DCs	(163)
	Reduced IL-18Rα expression	Genistein	*In vitro* human NK cell treatment/analysis	(167)
	Reduced IL-12/IL-18 dependent IFN-γ production	Genistein, daidzein, equol	*In vitro* pretreatment of cells, *in vivo* plasma cytokine measurement after treatment of mice	(167)
Macrophage	Decreased nitric oxide production	Genistein, daidzein	*In vitro* treatment of LPS-activated RAW 264.7 macrophages	(164, 168)
	Decreased iNOS expression	Genistein, diadzein	*In vitro* treatment of LPS-activated RAW 264.7 macrophages	(164, 168)
	Increased superoxide dismutase and catalase production	Genistein, daidzein	*In vitro* treatment of LPS-activated RAW 264.7 macrophages	(164, 169)
	Increased M2 polarization	Genistein	*In vivo* counting in various tissue after DSS-induced colitis and treatment in mice	(161)
	Increased ARG-1 and IL-10 expression in M2 macrophages	Genistein	*In vivo* characterization of mouse splenic M2 macrophages after DSS-induced colitis and treatment	(161)
Dendritic Cell	Inhibited cytokine secretion	Genistein, daidzein	*In vitro* treated LPS-activated monocyte-derived DCs	(163)
	Decreased TLR-dependent maturation marker expression	Genistein, daidzein	*In vitro* treated LPS-activated monocyte-derived DCs	(163)
	Decreased MHC class 1 expression	Genistein, daidzein	*In vitro* treated LPS-activated monocyte-derived DCs	(163)
	Inhibited CD4+ T-cell priming	Genistein, daidzein	*In vitro* co-culture of treated, activated monocyte derived DCs and naïve CD4 T-cells	(163)
	Increased ability to activate NK cells	Genistein, daidzein	*In vitro* co-cultures of treated, activated monocyte-derived DCs and autologous NK cells	(163)
Other granulocytes	Inhibited mast cell degranulation	Genistein, daidzein		(162, 164)

Interactions of the B-cell compartment with phytoestrogens are less well-characterized, but limited studies have reported that phytoestrogens can induce an anti-inflammatory, anti-allergic phenotype that could be beneficial for the host. Specifically, multiple studies have shown that isoflavones and coumestrol can lower serum titers of immunoglobulin G2a (IgG2a) antibodies (162–165). One such study also showed that low-dose coumestrol can decrease the titers of antigen-specific IgG1 and IgG3 during experimental autoimmune thyroiditis (165). Further isoflavones can suppress the expression of IgE, possibly thereby contributing to the overall anti-allergic phenotype that has been reported in response to phytoestrogen treatment in several animal models, including but not limited to airway allergy and peanut-sensitization models (162).

Phytoestrogens have also been shown to modulate the innate immune system and the majority of studies suggest an anti-inflammatory role in this context. Combination of isoflavones genistein and daidzein alone, or these plus glycitein have been shown to inhibit the ability of dendritic cells (DCs) to induce the production of IFN-γ, TNF-α, IL-9, and IL-13 from CD4+ T-cells (162, 163). These phytoestrogens have also been shown to inhibit direct cytokine secretion from activated DCs (163). Phytoestrogens also suppress DC maturation and the expression of MHCI, but not MHCII, in an intra-nasal allergic response model. These data suggest that phytoestrogens might slow the inflammatory immune response by inhibiting the antigen-presentation and effector-cell priming functions of DCs (162, 163). Genistein and daidzein, in particular, can suppress allergic inflammation by significantly reducing (by 25–30%) mast cell degranulation (162, 164). However, treatment of activated DCs with genistein or daidzein led to increased NK-cell degranulation and cytotoxicity, outcomes that have not been studied in a disease (163). Phytoestrogens can also modulate NK cell activity by specifically reducing expression of IL-18 receptor α (IL-18Rα), and inhibiting IFN-γ production in response to IL-12 and IL-18 (167). These actions of phytoestrogens have not been found to reduce NK cell cytotoxicity (156, 157, 163, 166). Thus, it remains unclear why phytoestrogens might have anti-inflammatory functions in DC populations, but potentially mixed effects in NK cell populations.

In macrophages, phytoestrogens have been shown to induce overall anti-inflammatory responses. Dia et al. showed that genistein and daidzein can decrease the production of nitric oxide and the expression of iNOS (inducible nitric oxide synthase), as well as inducing the activities of super oxide dismutase and catalase (168). Another study found that genistein treatment can skew macrophage polarization toward an M2, anti-inflammatory phenotype, while also reducing systemic concentrations of inflammatory cytokines (161). The same study also found that macrophages induced to take on the M2 phenotype when treated with genistein express ARG-1 and IL-10 at higher levels than those induced to take on this phenotype by other agents (161). Thus, genistein appears to push macrophages toward an actively anti-inflammatory phenotype, i.e., its actions are not solely non-inflammatory. The collective activities of the phytoestrogens in regard to the innate immune compartment may explain some of the systemic anti-inflammatory effects of phytoestrogens that have been described in the literature (e.g., decreased allergic responses and decreased autoreactive immune responses).

## Phytoestrogens and the Experimental Autoimmune Encephalomyelitis (EAE)

EAE is a very well-studied model of MS in which myelin antigen in combination with pertussis toxin and complete Freud's adjuvant is used to induce an autoimmune response. It is characterized by spinal cord pathology, manifesting in ascending paralysis that can be scored on a standard 6 point scale (0–5) (170). Several groups have documented the therapeutic and disease-preventative potential of phytoestrogens using the murine EAE model of MS. These studies indicate that common phytoestrogens, especially isoflavones genistein and daidzein, have potential as therapeutics for autoimmunity affecting CNS components. One group showed that sub-cutaneous (s.c.) treatment with genistein post-EAE induction resulted in significantly ameliorated EAE (171). The genistein treatment group showed reduced production of pro-inflammatory cytokines (including TNFα, IFNγ, and IL-12p40) by splenocytes and/or CNS lymphocytes (171). Similarly, 7-O-tetradecanoyl (TDG), a lipophilic genistein analog, suppressed disease when administered s.c. 14-days post-EAE induction. In these studies, disease amelioration correlated with a decrease in the number of IL-17 producing CD4+ T cells, and an increase in the number of FoxP3+CD4+ T cells, in the brain (172). Another study reported that daily oral treatment with high-dose daidzein starting 10 days after EAE induction ameliorated disease and simultaneously reduced IFNγ levels in the brain and splenocytes compared to controls with induced disease (173). These studies suggest that phytoestrogenic compounds or their analogs might have therapeutic potential in an animal model of MS.

Besides isoflavones, other phytoestrogens have also been shown to exert a protective effect in EAE. Wei et al. showed that therapeutic oral administration of high-dose Icariin (ICA), a phytoestrogen from flowering plants of the Epimedium genus, ameliorated EAE (comparison was to vehicle control). The effectiveness of ICA was similar to that of estrogen, and high-dose ICA or estrogen treatment increased expression of both ERα and ERβ in the white matter of the CNS (174). In a separate study, these researchers reported that therapeutic oral administration of high-dose ICA in combination with methylprednisolone (MP), a corticosteroid used therapeutically in MS, had a greater disease-ameliorating effect than either treatment alone. This combination treatment (ICA+MP) also had a synergistic effect, enhancing both the reduction of serum IL-17 and apoptotic cell death (Annexin V^+^ cells) in the spinal cord (175). Taken together, these studies show the therapeutic potential of phytoestrogen compounds, both alone and in combination therapies, as a promising complementary and alternative therapy for further study.

Quercetin is a phytoestrogen flavonoid that is abundant in soybeans, vegetables, and fruits and has also been evaluated for its efficacy in the EAE model. Quercetin protects against EAE when injected intra-peritoneally (i.p.; comparison was to a vehicle control). Additionally, quercetin caused a dose dependent suppression of antigen specific proliferation and IL-12 in splenocytes in *ex-vivo* antigen-recall response (176). Thus, quercetin might be able to influence encephalitogenic T cells directly. Resveratrol, a phytoestrogen found in the skins of red grapes and berries, had been shown to ameliorate EAE by interfering with the miR-124/sphingosine kinase 1 (SK1) axis in encephalitogenic T cells, thereby resulting in cell-cycle arrest and apoptosis (177). These studies clearly indicate that phytoestrogen compounds protect against EAE and may have implications/therapeutic potential in MS as well.

However, several knowledge gaps remain to be addressed. For example, most of the studies described above introduced phytoestrogen compounds via a non-physiological route (s.c. or i.p.). Given that humans obtain phytoestrogens through diet, study of the effectiveness of oral delivery or consumption of these compounds would better reflect the mechanisms involved in a more physiological context. Furthermore, the studies using s.c. or i.p. routes of administration do not account for the importance of phytoestrogen-metabolizing gut bacteria, which humans rely on for proper breakdown of dietary phytoestrogens. This may explain why the studies that did provide oral phytoestrogens required a very high dose for protection.

Although the exact mechanism through which phytoestrogenic compounds suppress EAE is unknown, studies have suggested that their neuroprotective and immunomodulatory effects might play an important role in their ability to suppress disease as described above.

## Concluding Remarks

In RRMS patients, the presence of gut dysbiosis and the depletion of bacteria with the ability to metabolize phytoestrogens highlights the importance of these compounds in maintaining a disease free-state of the host. As stated above, various phytoestrogen metabolites play important roles in a number of biological processes including neuroprotection and regulation of the immune system. However, further research is certainly needed to better understand the pathways through which gut bacteria induced phytoestrogens metabolites regulate the balance between pro- and anti-inflammatory responses and provide neuroprotection. For example, future studies determining the relationship between levels of phytoestrogen metabolites and the severity of RRMS disease are expected to shed light on the extent to which phytoestrogen metabolism correlates with the etiopathogenesis of RRMS. Also, dissection of the role of phytoestrogen metabolism in the development and regulation of the immune system in germ-free mice is expected to reveal the significance of specific phytoestrogen metabolites in regulating the function of various immune subsets. In the meantime, however, the existing literature provides a solid rationale for the selection and testing of the therapeutic potential of various phytoestrogen metabolizing bacteria, including *Prevotella, Parabacteroides, and Adlercreutzia* in a preclinical model of MS. A successful outcome from these studies will help in development of bacteria as drug (BRUG) based treatment options for MS patients.

## Author Contributions

AM conceptualized the review, helped with overall structure of the manuscript, and gave final approval of the manuscript to be published. NC wrote the phytoestrogen metabolism section of the manuscript. SF wrote role of phytoestrogen in EAE and nervous system section. SP helped with writing the section on phytoestrogen receptors and effect of phytoestrogen on immune cells. All authors commented on the manuscript.

### Conflict of Interest

AM is one of the inventor of a technology claiming the use of *Prevotella histicola* for the treatment of autoimmune diseases. The patent for the technology is owned by Mayo Clinic, who has given exclusive license to Evelo Biosciences. AM received royalties from Mayo Clinic (paid by Evelo Biosciences). The remaining authors declare that the research was conducted in the absence of any commercial or financial relationships that could be construed as a potential conflict of interest.
